# Stigma, Perceived Discrimination, and Mental Health during China’s COVID-19 Outbreak: A Mixed-Methods Investigation

**DOI:** 10.1177/00221465211040550

**Published:** 2021-10-04

**Authors:** Wen Fan, Yue Qian, Yongai Jin

**Affiliations:** 1 Boston College, Chestnut Hill, MA, USA; 2 University of British Columbia, Vancouver, BC, Canada; 3 Renmin University of China, Beijing, China

**Keywords:** China, COVID-19, discrimination, mental health, stigma

## Abstract

Research on stigma and discrimination during COVID-19 has focused on racism and xenophobia in Western countries. In comparison, little research has considered stigma processes, discrimination, and their public health implications in non-Western contexts. This study draws on quantitative survey data (N = 7,942) and qualitative interview data (N = 50) to understand the emergence, experiences, and mental health implications of stigma and discrimination during China’s COVID-19 outbreak. Given China’s history of regionalism, we theorize and use a survey experiment to empirically assess region-based stigma: People who lived in Hubei (the hardest hit province) during the outbreak and those who were socially associated with Hubei were stigmatized. Furthermore, the COVID-19 outbreak created stigma around people labeled as *patients* by the state. These stigmatized groups reported greater perceived discrimination, which—as a stressor—led to psychological distress. Our interview data illuminated how the stigmatized groups perceived, experienced, and coped with discrimination and stigma.

As the COVID-19 pandemic spreads around the globe, discrimination against Asians has surged (Tessler, Choi, and Kao 2020). Viewed as the physical embodiment of the disease, Asians living in the West have been “stabbed, beaten, bullied, spit on, pushed, harassed, and vilified based on the false assumption that they are to blame for the spread of COVID-19” (Lee and Yadav 2020:17). To date, research on the experiences and health consequences of discrimination and stigma during COVID-19 has focused on racism and xenophobia in Western countries (e.g., Lee and Yadav 2020; Tessler et al. 2020; C. Wu, Qian, and Wilkes 2021). In comparison, little empirical research has investigated stigma processes and their public health implications in non-Western contexts.

This study focuses on the context of China, where the COVID-19 outbreak first occurred. Different from many Western countries, racial and nativity differences are not as salient in China given that its population overwhelmingly consists of native-born ethnic Chinese (X. Wu and He 2016). What human differences, if any, emerged as the basis for stigma and discrimination during China’s COVID-19 outbreak? The social production of stigma framework (Link and Phelan 2001) leads us to theorize that, facilitated by institutional power, the outbreak has activated and exacerbated certain forms of stigma that existed in China long before the crisis while producing new forms of stigma directly related to COVID-19. Specifically, given the long history of regionalism in China (Eberhard 1965; Moser 2019), we expect region-based differences to be amplified and become a basis for stigma during COVID-19. Thus, people who lived in Hubei (the hardest hit province) during the outbreak or those who were socially associated with Hubei were likely stigmatized. Additionally, the COVID-19 outbreak may have triggered stigma around people labeled as *patients*. The perceived discrimination experienced by these stigmatized groups—as a stressor—may lead to mental distress (Pearlin et al. 2005) yet also to creative adoption of various coping strategies to manage such distress (Link and Phelan 2013). These processes are shown in Figure 1.

**Figure 1.**
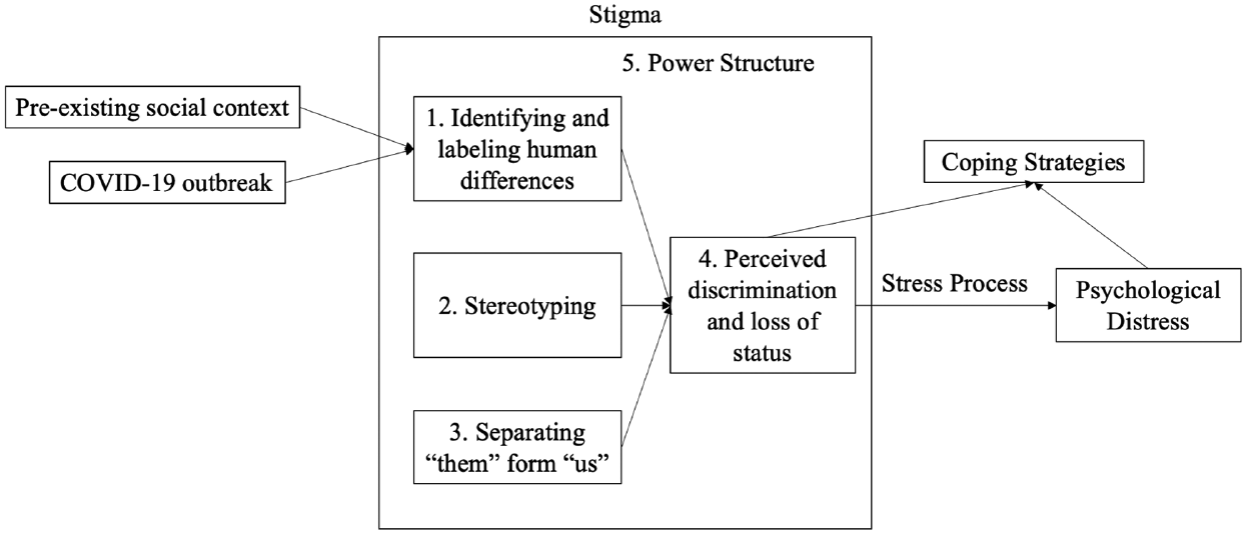
Conceptual Model.

To understand the emergence, experiences, and well-being implications of stigma and discrimination around COVID-19, between March and May 2020, we collected national survey data from 7,942 Chinese respondents and interview data from 50 Wuhan residents (Wuhan is the capital city of Hubei province). We first draw on an experiment embedded in the survey to assess stigma associated with “Hubeiness.” We then use survey data to show that perceptions of discrimination were disproportionally borne by people who lived in or were socially associated with Hubei and people who were once suspected or confirmed to have COVID-19. Survey data are also used to quantify the relationship between perceived discrimination and psychological distress and the role of perceived discrimination in accounting for the worse well-being of people hit hardest by the COVID-19 outbreak. Finally, we use in-depth interview data to illustrate how Wuhan residents perceived, experienced, and reacted to discrimination and stigma.

We make several contributions to the literature. First, our research is among the first to empirically demonstrate the existence of region-based stigma in China during COVID-19. To get at the prevalence and nature of stigma, researchers typically ask individuals about their experiences with discrimination using surveys or interviews (Krieger 2000). We collected not only these data but also experimental data on Chinese respondents’ conceptions of Hubei and another otherwise similar province. Results thus provide robust evidence on the existence of stigma and offer insights into the coherence between actual and perceived discrimination. Second, our research advances understanding of the structural aspect of stigma by illuminating how larger social and institutional processes generate and intensify stigma and discrimination in a crisis setting (Link and Phelan 2001; Mahajan et al. 2008; Parker and Aggleton 2003). Specifically, we show how institutional power, in the forms of state policies and structural forces, amplifies certain group differences and makes culturally created and disease-associated categories relevant for producing stigma. Third, we reveal the critical role of perceived discrimination in the psychological distress borne by people geographically or socially associated with the epicenter as well as people infected with COVID-19. Coupled with recounts of how the stigmatized groups actively coped with such distress, our research contributes much needed knowledge to designing (post)pandemic recovery interventions. Lastly, more broadly, our study contributes to discrimination research by demonstrating the health-damaging implications of discrimination, thereby producing knowledge useful for promoting social well-being.

## Background

### Stigma Theory

Stigmatization is a process of devaluing individuals who possess “an attribute that is deeply discrediting” (Goffman 1963:3). Through stigmatization, individuals are systematically excluded from social interactions because they possess a characteristic that is perceived to violate society’s normative expectations or endanger others’ health and safety (Corrigan et al. 2002; Pescosolido et al. 1999). To develop an analytic framework, Link and Phelan (2001:377) conceptualize that stigma involves “labeling, stereotyping, separation, status loss, and discrimination . . . in a power situation that allows them” (see the “Stigma” box in Figure 1). Specifically, people first identify and label human differences. Then, stereotyping occurs, linking the labeled person to undesirable characteristics. A third element involves the separation of “them” (the stigmatized group) and “us” (the group doing the labeling). Combined, stigmatized people experience status loss and discrimination. Finally, stigmatization is contingent on the use of social, economic, and political power that allows labeling, stereotyping, separation, status loss, and discrimination to unfold. Unlike a psychologically oriented lens, Link and Phelan’s model is deeply sociological. Which human differences are deemed relevant and become a source of stigma are largely socially constructed; moreover, power is essential to stigma production (Link and Phelan 2001, 2013).

Guided by Link and Phelan’s (2001) model, we examine the structural conditions that produce and reinforce stigma. Two decades ago, when writing on the HIV/AIDS epidemic, Parker and Aggleton (2003) argued that stigma functioned at the intersection of power, culture, and difference, and they called for greater attention to the broader political economy of HIV/AIDS-related stigma. Subsequent work showed that laws and policies that criminalized consensual homosexual activity, prohibited needle exchange, and required proof of residency status to access services all contributed to the stigmatization of HIV/AIDS (for a review, see Mahajan et al. 2008).

Therefore, examining stigma within a structural framework necessitates a focus on power—social, economic, or political—to understand the processes leading to stigmatization and social exclusion of certain groups. In this research, we reveal how state policies and institutions, facilitated by technology and social media, contributed to stigma production in a public health crisis. Next, we discuss two types of stigma that we expect to emerge during China’s COVID-19 outbreak.

### Disease-Associated Stigma during China’s COVID-19 Outbreak

We expect stigma to arise around people suspected or confirmed to have COVID-19. Some marks, particularly those perceived to endanger public health, tend to evoke fear and stigmatization, with greater fear associated with stronger social rejection (Corrigan et al. 2002; Pescosolido et al. 1999). Kurzban and Leary (2001:192), for example, provided a functional explanation of stigma, arguing that stigma derives partly from “parasite avoidance” (i.e., the evolutionary adaptations to prevent contact with those who may carry communicable pathogens). Similarly, Phelan, Link, and Dovidio (2008) posited that one function of stigma is to “keep people away.” Indeed, throughout history, humans have coped with their fear in regard to contagious disease by blaming and ostracizing the “other” (Joffe 1999). This parasite avoidance tendency may be particularly strong during China’s COVID-19 outbreak because China was the first community exposed to this highly infectious new disease that still lacks effective treatment. Combined with the human tendency to be biased toward false positives when it comes to survival (Kurzban and Leary 2001), COVID-19-related stigma likely emerges, targeting even recovered patients who pose no actual threat.

Stigma thus likely arises around “COVID-19 cases,” but how is infection status, which is arguably concealable, known by others? In the pandemic time of heightened public disclosure of contact tracing data and increased surveillance, individuals’ ability to keep their health status private may well be undermined (Singer and Sang-Hun 2020). This was true in China where the exercise of institutional power in COVID-19 containment efforts may have inadvertently bridged the gap between public stigma (i.e., stereotypes and discrimination against a social group endorsed by the general population) and discrimination experienced by actual individuals.

To curb the outbreak, starting in February 2020, China conducted community-based, door-to-door screening across the country and enforced strong measures to ensure that confirmed patients and suspected patients (i.e., those who exhibited COVID-19 symptoms but had not been clinically diagnosed) were transferred to designated health care facilities for hospitalization, treatment, or quarantine (State Council Information Office 2020). Who were sent to the collective facilities might be known by neighbors given China’s high-density residential complexes. Additionally, *shequ* (community residence committees), functioning as the lowest level government in China, were required to provide residents with updated information on COVID-19 cases in their neighborhood (Qian and Hanser 2021). Such information was typically announced in chat groups on WeChat (China’s most popular social media platform). Neighborhood WeChat groups existed even before COVID-19, but they became almost universal during COVID-19 to facilitate coronavirus surveillance and public service provision. Although releasing patients’ personal information was forbidden, WeChat groups could be a gossip mill as neighbors exchanged information there to guess and pinpoint who was infected (Wang 2020). These practices increased the chances that one’s COVID-19 infection status became public knowledge and thus a basis for discrimination.

*Hypothesis 1:* Individuals who were confirmed or suspected to have COVID-19 were more likely to perceive discrimination.

### Region-Based Stigma during China’s COVID-19 Outbreak

During COVID-19, those who are socially associated with the virus may also be stigmatized and discriminated against. But which dimension of human differences invokes stigma? We argue that precrisis social organizations and long-existing societal fissures shape the forms of stigma and discrimination in a crisis.

In China, region is widely used to categorize people; region-based stereotyping, colloquially referred to as *diyu hei* in modern Chinese, has existed for over 2,000 years (Eberhard 1965; Moser 2019). Moreover, no institutional protection exists for people who are discriminated against based on region (*The Economist* 2019). Categorizing people based on region thus provides a readily available template in the COVID-19 crisis, which likely activates and exacerbates region-based stigma. Given that the COVID-19 outbreak first occurred in Hubei, which had over 80% of all COVID-19 cases in China (China Data Lab 2020), we expect that people who either lived in Hubei during the peak of COVID-19 or originated from Hubei stand a high chance of experiencing discrimination.

Social media play an important role in transmitting stigmatizing messages, especially for Hubei residents. Given the lockdown in Hubei between January and April 2020, movement of people in and out of their province, city, or even residential compound was strictly controlled (State Council Information Office 2020). Thus, the type of discrimination Hubei residents experienced was unlikely to be face-to-face. Tensions were, however, already building up on social media sites, with Hubeiness being blamed for producing and spreading the coronavirus (He et al. 2020). Furthermore, attitudes toward Hubeiness quickly geared from COVID-19 to stereotyping and moral transgressions such as eating wild animals (Sixiangchao 2020).

Compared with Hubei residents, Hubei-origin people living outside Hubei were more likely to experience in-person discrimination. *Hubeiness* became a salient label because local governments outside Hubei focused efforts on finding and isolating migrants who were socially associated with Hubei (e.g., with a Hubei *hukou*) even after they were tested negative (He et al. 2020; Mozur 2020). This was facilitated by the *hukou* (household registration) system, which assigns each citizen a registered residential place at birth; *hukou* does not change automatically with geographical mobility and is needed for almost everything, including entering school, accessing welfare, and registering marriage, effectively a tool for migration control and mass surveillance (Chan 2009). The citizen identity card—which contains information on places of birth—is another way for one’s origins to be known by others. Because a citizen identity card is needed for checking into hotels, news reports abound that Hubei-origin people were denied access to accommodations regardless of their health conditions (He et al. 2020; Mozur 2020).

Given these processes, we expect that Hubeiness invoked public stigma and became a basis for discrimination during China’s COVID-19 outbreak. Additionally, we expect:

*Hypothesis 2:* People who lived in Hubei during China’s COVID-19 outbreak and Hubei *hukou* holders who lived outside Hubei were more likely to perceive discrimination.

### Perceived Discrimination, Mental Health, and Coping Strategies

Perceived discrimination has been an important topic in mental health studies (Krieger 2000). From a stress process perspective (Pearlin et al. 2005), being stigmatized and the perception of discrimination that follows represent a source of stress, which can lead to negative effects on mental health. Through socialization and daily observation, many Chinese people adopt internalized beliefs about how regional labeling works and how people with contagious diseases are treated. When the social group they belong to—in this case, Hubeiness or suspected/confirmed COVID-19 patients—is set apart and linked to undesirable characteristics, these stigmatized individuals realize that a negative label has been applied to them (Link et al. 1989; Link and Phelan 2001). The perceived devaluation, rejection, and social exclusion likely threaten the stigmatized individuals’ needs for acceptance and inclusion (Tajfel and Turner 2004). Furthermore, discrimination delivers the message that the targeted individuals are unworthy and dangerous, which in turn could undermine mental well-being (Goffman 1963; Link and Phelan 2001). Consistent with these arguments, a large body of empirical evidence shows that perceived discrimination is a dire psychosocial stressor that is associated with negative psychological well-being outcomes (Kessler, Mickelson, and Williams 1999; Krieger 2000; Noh et al. 1999; Williams and Mohammed 2013; C. Wu et al. 2021).

Building on existing studies of discrimination and mental health, we investigate whether perceptions of discrimination lead to psychological distress during China’s COVID-19 outbreak. It was a time when the labels of *Hubeiness* or *suspected/confirmed COVID-19 patients* were no longer objective descriptions but imbued with stereotypical beliefs about the dangerousness of labeled persons. Considering that these labeled persons likely felt not only devalued and rejected but also falsely accused, perceived discrimination can be especially harmful for the psychological well-being of the stigmatized people in the COVID-19 context.

*Hypothesis 3:* Individuals with higher perceived discrimination experienced greater psychological distress during China’s COVID-19 outbreak.

Furthermore, we expect perceived discrimination to constitute a mechanism that explains the potentially poorer psychological well-being of Hubeiness and confirmed/suspected COVID-19 patients. An often investigated question in the mental health literature is to what extent discrimination explains the mental health disparities between advantaged and disadvantaged social groups, such as between men and women or between whites and racial minorities (Kessler et al. 1999; C. Wu et al. 2021). We follow this line of inquiry to argue that perceived discrimination represents a major stressor that can account for the disparities in mental health across social groups who are differentially affected by the pandemic.

*Hypothesis 4:* Perceived discrimination mediates the relationship between Hubeiness and psychological distress and the relationship between COVID-19 infection status and psychological distress.

Individuals are nevertheless not merely passive victims, but they actively respond to social dislocations with creative strategies. In the face of stigma, different coping strategies may be used, such as *secrecy* (concealing labeling information), *withdrawal* (avoiding potentially rejecting situations), *education* (providing information to counter stereotypes), and *challenging* (directly confronting stigmatizing behavior; Link et al. 1989; Link and Phelan 2013). Drawing on in-depth interview data, our last research aim is to identify strategies used by the stigmatized groups to manage stigma, discrimination, and resulting psychological distress during China’s COVID-19 outbreak.

## Data And Methods

### Data

This mixed-methods study drew on original data from a national survey and in-depth interviews. Both the survey and the interviews belonged to a larger project on Chinese people’s experiences and mental health during COVID-19. Due to COVID-19 travel restrictions, the survey and interviews were conducted online.

#### Survey sample

Between March 20 and April 29, 2020, an online survey was conducted in Mainland China by a professional survey firm. We oversampled Hubei residents because Hubei was hit hardest by COVID-19. Within each stratum (Hubei, other provinces), a quota was set based on gender, age, and education to ensure sample diversity (see also Qian and Fan 2020). Some respondents were from the firm’s existing research panel, prerecruited through various online and offline channels. Additional respondents (mostly oversampled Hubei residents) were recruited specifically for this project via local university-based research networks, social media, and various Hubei-based websites. To ensure data quality, protections against bots or multiple completions from the same IP address were implemented; the survey also included attention check questions—only panelists who passed at least half of these items were kept; 7 to 10 additional verifications (e.g., consistency between reported age group and year of birth) were applied to screen out panelists failing over half of these checks. Respondents were compensated 10 RMB (roughly $1.50) for completing the survey.

**Figure 2.**
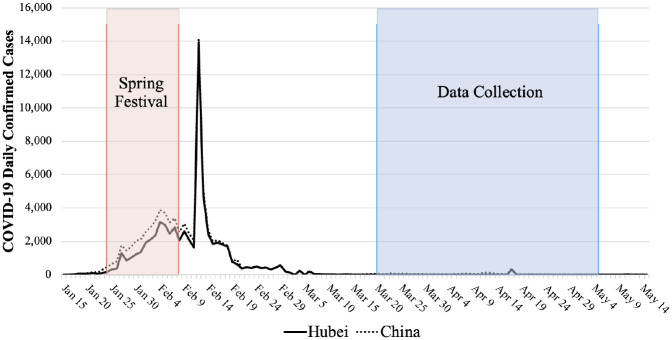
COVID-19 Daily Confirmed Cases in China, January 15 through May 15, 2020. *Source:* China Data Lab (2020). ^a^Our survey and interview data were collected after the peak of China’s COVID-19 outbreak. A few measures in the survey, such as perceived discrimination and psychological distress, were retrospective (referring to the 2020 Spring Festival period). The interview data collected detailed information on interviewees’ experiences, challenges, and coping strategies from early 2020 to the interview date. ^b^The surge of new cases on February 12, 2020, was due to change in how cases were diagnosed and reported. Starting on February 12, in Hubei province, chest imaging alone was sufficient to classify a suspected case of COVID-19 as clinically confirmed (as opposed to having to have a laboratory confirmation).

The final sample included 5,010 adults who lived in Hubei province and 3,000 adults who lived in other provinces during the Chinese Spring Festival (January 24 to February 8, 2020). The Spring Festival is the grandest festival in China; in 2020, it coincided with the period when the number of COVID-19 patients was surging (Figure 2). Thus, occurrences and experiences during this period would be particularly memorable to respondents. After excluding 68 respondents (.85%) with missing data on variables used, our analytic sample consisted of 7,942 respondents.

Although we were cautious about generalizing given the opt-in nature of the sample, the survey data allowed us to provide an account of disparities in perceived discrimination across social groups and the relationship between perceived discrimination and mental health. Additionally, it is worth noting that disaster research faces many challenges in data collection. Perishable data (data that will change or get lost over time) are nevertheless valuable in recording activities in context and documenting individual accounts while the memory is still fresh (Oulahen, Vogel, and Gouett-Hanna 2020). A convenience sample is often necessary to collect these perishable data (Goldmann and Galea 2014).

#### Interview sample

Between March 27 and May 5, 2020, the second author and two research assistants conducted semistructured interviews with 50 people who lived in Wuhan during the outbreak. Wuhan, where the COVID-19 outbreak first occurred, is the capital city of Hubei province. Interviewees were recruited through personal connections, social media, and snowball sampling. In the interviews, we asked open-ended questions about participants’ experiences, challenges, and coping strategies during the outbreak. Interviews lasted from 51 to 192 minutes (mean = 109 minutes). All interviews followed standard consent and confidentiality protocols, as approved by the research ethics board at the second author’s institution.

Interviewees’ age ranged from 21 to 65 years (mean = 35 years), and education ranged from junior high school education to doctoral degrees. There were 11 men and 39 women. Women were overrepresented because one project aim was to understand, through interviews, the experiences of people with special health care/caregiving needs during COVID-19. Pregnant women and parents of young children (women are often the primary caregiver of young children in China) were therefore two of the target interview groups. Our analysis of the interviews suggested that the qualitative themes were similarly revealed by both men and women.

### Survey Measures

#### Social distance

To gauge stigma associated with Hubeiness, we adopted measures of social distance, a widely used measure of stigma that is applicable to the adult population (Link et al. 2004). To facilitate causal inference, we used a survey–experimental design. Two social distance questions were presented: “Nowadays, how do you think most people in this society would feel about having . . . enter the city (or enter the neighborhood) they live in?” Through randomization, about half of the respondents were asked about Sichuan people (*Sichuan ren*), and the other half were asked about Hubei people (*Hubei ren*; for additional details on the survey experiment items, see Appendix Section 1 in the online version of the article). By assessing the extent to which individuals believed that “most people” (the society at large) would distance themselves from a certain group (i.e., Sichuan people or Hubei people), these questions captured the stigma around that group while minimizing social desirability bias (i.e., unwillingness to voice socially undesirable attitudes; Link et al. 1989, 2004). Each item was rated on a 4-point Likert scale (very willing, somewhat willing, somewhat unwilling, very unwilling). Also note that in Chinese, “Sichuan/Hubei people” can be interpreted as people living in or originating from Sichuan/Hubei.

We chose Sichuan as a control group because it is comparable to Hubei in many regards. The two provinces are similar in population size, urbanization rate, and economic development (GDP per capita); they are geographically close and share similar dialects that belong to one of China’s eight major dialect groups. Additionally, Hubei and Sichuan are generally not associated with negative preexisting stereotypes. Thus, respondents’ attitudes toward Sichuan people could be a reasonable proxy for their attitudes toward Hubei people in the absence of COVID-19.

#### Perceived discrimination

We measured perceived discrimination through the question: “How often did you feel being discriminated against during the 2020 Spring Festival?” Frequency was reported as never (0), rarely (1), sometimes (2), and often/always (3; often and always were combined because of small sample sizes).

#### Psychological distress

The 10-item version of the Center for Epidemiologic Studies Depression Scale was used. Respondents were asked whether during the 2020 Spring Festival they (1) were bothered by things that usually don’t bother them, (2) had trouble keeping their mind on what they were doing, (3) felt depressed, (4) felt that everything they did was an effort, (5) felt hopeful about the future, (6) felt fearful, (7) felt that their sleep was restless, (8) were happy, (9) felt lonely, and (10) felt that they could not “get going.” Each item was rated on a 4-point scale, ranging from 0 (rarely or none of the time) to 3 (most or all of the time). We reverse-coded the two positive mood items and calculated the sum of the 10 items (α = .86; range = 0–30).

#### COVID-19 infection status

Respondents were asked whether they had ever been suspected or confirmed COVID-19 patients (nonpatient, patient, prefer not to answer). We combined suspected (n = 90) and confirmed (n = 26) patients into one group because of the small sample sizes and because both groups were classified as “vulnerable populations” in China (State Council Information Office 2020). People who preferred not to indicate their infection status were not our focus but were included as a separate group in our analysis.

#### Hubeiness

We defined Hubeiness based on respondents’ geographical or social associations with Hubei. Specifically, we classified respondents into three categories: Hubei residents (who lived in Hubei during the Spring Festival, n = 4,985), Hubei people living outside Hubei (who held a Hubei *hukou* but did not live in Hubei during the Spring Festival, n = 59), and non-Hubei people (who lived outside Hubei with a non-Hubei *hukou*, n = 2,898).

#### Control variables

We controlled for age and educational attainment (less than high school, high school, junior college, and university or above) because older people and those with less education were underrepresented in our sample (relative to representative samples). We also controlled for potential confounders associated with mental health and perceived discrimination (Kessler et al. 1999; Krieger 2000). Demographic covariates included gender (1 = female, 0 = male), marital status (never married, married, and previously married), and presence of children in the household (no minor children, youngest child under 6 years, youngest child ages 6–17 years). Indicators of socioeconomic status included employment status prior to the outbreak (employed, unemployed, out of the labor force), rural (1) or urban (0) *hukou*, and monthly family income in 2019 (<5,000 yuan; 5,000–9,999 yuan; 10,000–19,999 yuan; ≥20,000 yuan). We also controlled for self-rated health prior to the outbreak (1 = fair or poor, 2 = good, 3 = very good, 4 = excellent). Table 1 shows descriptive statistics for all variables.

**Table 1.** Descriptive Statistics.

	mean or %	SD	Sample Size (*n*)
Perceived discrimination			
Never	43.60%		3,463
Rarely	32.59%		2,588
Sometimes	17.99%		1,429
Often/always	5.82%		462
Psychological distress	8.18	5.45	7,942
COVID-19 infection status			
Nonpatients	97.04%		7,707
Patients (suspected/confirmed)	1.46%		116
Prefer not to say	1.50%		119
Hubeiness			
Non-Hubei people	36.49%		2,898
Hubei residents	62.77%		4,985
Hubei people living outside Hubei	.74%		59
Female	50.06%		3,976
Age	31.02	9.61	7,942
Marital status			
Never married	48.12%		3,822
Married	49.77%		3,953
Previously married	2.10%		167
Presence of child			
No minor children	49.92%		3,965
Youngest child < 6	29.93%		2,377
Youngest child ages 6–17	20.15%		1,600
Education			
Less than high school	6.70%		532
High school	18.21%		1,446
Junior college	24.60%		1,954
University or above	50.49%		4,010
Employment status prior to the outbreak			
Employed	59.24%		4,705
Unemployed	13.70%		1,088
Not in the labor force	27.06%		2,149
Rural *hukou*	43.16%		3,428
Monthly family income in 2019			
<5,000 yuan	29.80%		2,367
5,000–9,999 yuan	33.27%		2,642
10,000–19,999 yuan	24.77%		1,967
≥20,000 yuan	12.16%		966
Self-rated health	3.05	.93	7,942

### Statistical Approach

We first examined percentage distributions of social distance measures to assess stigma associated with Hubeiness. We then used ordered logit models to examine correlates of perceived discrimination, with a focus on the roles of Hubeiness and COVID-19 infection status. Next, we used ordinary least squares regression models to investigate whether perceived discrimination was associated with psychological distress and whether perceived discrimination accounted for disparities in psychological distress by region or COVID-19 infection status. For all statistical analyses, we used nonparametric permutation tests to determine the significance of coefficients. We ran analysis with 10,000 permutations following convention. Permutation tests are often used for inference when assumptions required for parametric inference are unmet, as in our case of a nonprobability sample (Good 2013). Permutation tests are particularly useful when sample sizes are small, and they are more robust than their parametric counterparts (Good 2013). The permutation *p* value indicates how likely each coefficient could have been as extreme as the observed value by chance alone.

### Interview Coding and Analysis

We followed a three-stage process for analyzing interview data (Deterding and Waters 2021). First, assisted by MAXQDA, the second author checked the transcripts for accuracy and familiarized herself with all aspects of the data (Nowell et al. 2017). Although we did not set out to study experiences of discrimination and stigma, it emerged as an important theme: 30 out of 50 interviews involved discussions around this theme. After line-by-line deep readings of the transcripts, the second author indexed transcripts to identify large chunks of text related to stigma and discrimination. Next, she applied analytic codes to systematically code indexed extracts in an iterative fashion. Analytic codes included both theoretically established deductive codes (e.g., “region-based discrimination,” “disease-associated discrimination,” “emotions”) and inductive codes emerged from the interviews (e.g., “coping strategies”; Deterding and Waters 2021). Third, to check intercoder reliability, the first author coded all excerpts independently and compared with the second author’s codes to ensure agreement on data interpretation. In the writing process, we also made sure that our conceptualization, interpretation, and translation represented interviewees’ views in an accurate way (Nowell et al. 2017). Pseudonyms are used in the following to maintain anonymity.

## Results

### Survey Results

#### Survey experiment

**Figure 3.**
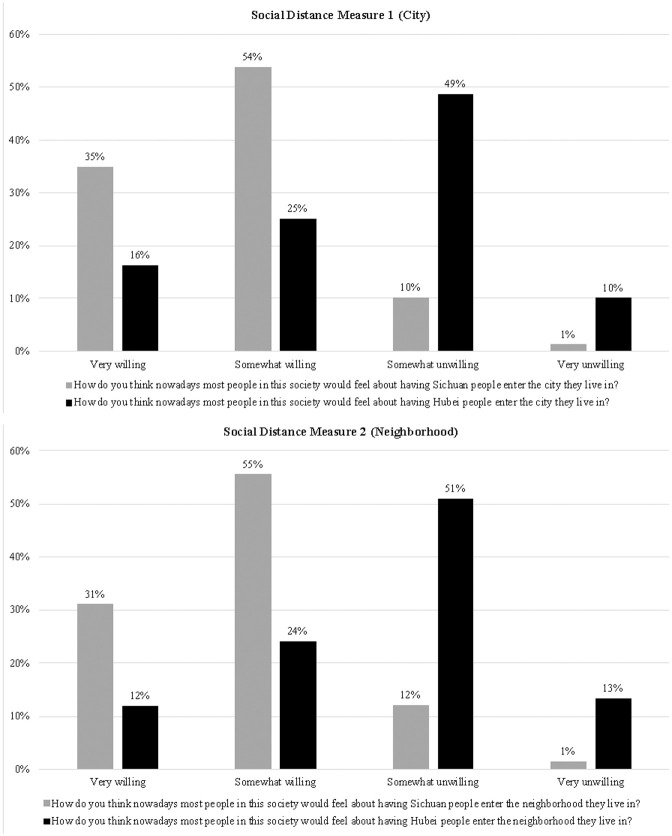
Percentage Distribution of the Responses to Social Distance Measures in Survey Experiment.

In Figure 3, we present the percentage distribution of responses to social distance measures. Recall that respondents were randomly assigned to answer two questions pertaining to either Sichuan people or Hubei people. Because of the similarities between Sichuan and Hubei before COVID-19 (as discussed previously), attitudes toward Sichuan and Hubei people should have been similar had the COVID-19 outbreak not occurred.

The difference in attitudes toward the two groups, however, could not be starker. Only 11% of respondents thought that most people would be either somewhat or very unwilling to have Sichuan people enter their city, but 59% of respondents thought that most people would be unwilling to have Hubei people enter their city. The result is similar when entering the neighborhood is examined. Only 13% of respondents thought that most people would be unwilling to have Sichuan people enter their neighborhood, but 64% of respondents thought that most people would be unwilling to have Hubei people as neighbors. Permutation chi-squared tests indeed show that the distributions of attitudes toward Sichuan and Hubei people are significantly different (*p* < .001 in both cases). These differences captured by the social distance measures likely reflect stigma, as opposed to legitimate fear of transmission, because these measures assessed public attitudes toward Hubei/Sichuan people at the time of the survey when few new cases were reported in Hubei (see Figure 2). Supplementary analysis indicated that Hubei people and non-Hubei people shared the belief that most people would distance themselves from Hubei (but not Sichuan) people (see Appendix Figures 1 and 2 in the online version of the article), which confirmed that both the stigmatized group and those who stigmatize are usually aware of the stigma and expect the stigmatized group to be rejected (Link et al. 1989). Thus, the survey experiment shows strong stigma attached to Hubeiness, which in fact became part of lay knowledge shared by both the stigmatized and those who stigmatize.

#### Correlates of perceived discrimination

In Table 2, we use ordered logit models to examine whether the two stigmatized groups—Hubeiness and suspected/confirmed COVID-19 patients—perceived higher discrimination during the Spring Festival. The short answer is yes, regardless of excluding or including covariates.

**Table 2.** Ordered Logit Models Predicting Perceived Discrimination, in Log Odds.

	Model 1		Model 2	
	Coefficient	Permutation *p* Value	Coefficient	Permutation *p* Value
COVID-19 infection status				
Nonpatients				
Patients (suspected/confirmed)	1.019	.000	.880	.000
Prefer not to say	.473	.005	.333	.057
Hubeiness				
Non-Hubei people				
Hubei residents	.762	.000	.749	.000
Hubei people living outside Hubei	1.014	.000	1.127	.000
**Control variables**				
Female			–.126	.003
Age			–.012	.000
Marital status				
Never married				
Married			–.082	.195
Previously married			.074	.629
Presence of child				
No minor children				
Youngest child < 6			–.007	.897
Youngest child ages 6–17			–.114	.050
Education				
Less than high school				
High school			–.276	.004
Junior college			–.237	.015
University or above			–.161	.089
Employment status prior to the outbreak				
Employed				
Unemployed			.254	.000
Not in the labor force			–.194	.000
Rural *hukou*			–.067	.149
Monthly family income in 2019				
<5,000 yuan				
5,000–9,999 yuan			.015	.785
10,000–19,999 yuan			.023	.704
≥20,000 yuan			.058	.436
Self-rated health			–.504	.000
Constant (cut1)	.235	.786	–2.051	.000
Constant (cut2)	1.706	.000	–.499	1.000
Constant (cut3)	3.358	.000	1.206	1.000

*Note: N* = 7,942. Interpretation of permutation *p* value: If 50 out of 10,000 permutations yield regression coefficients as large as the observed value, the probability that the actual coefficient could be the result of random sampling error is about .005.

**Figure 4.**
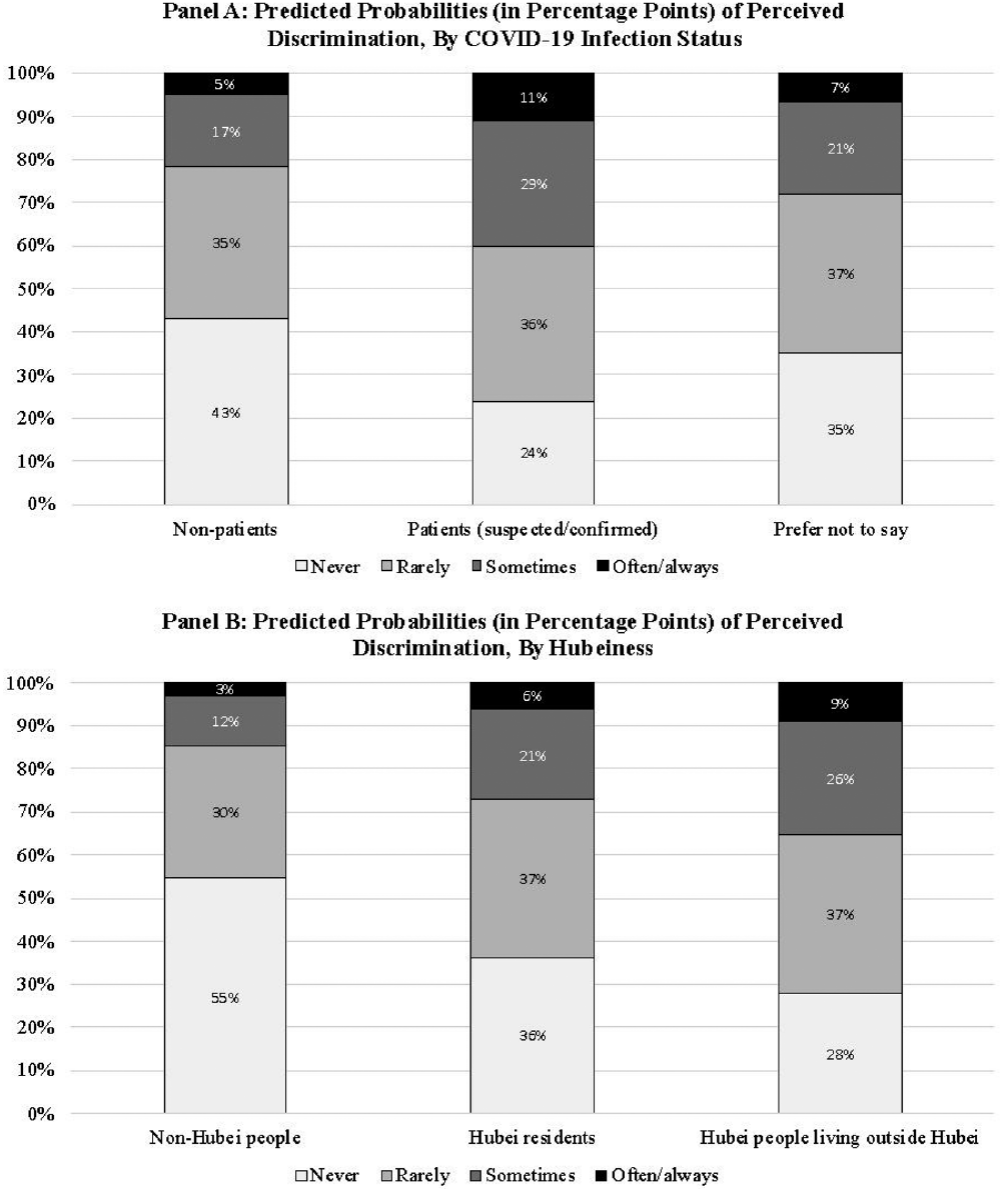
Predicted Probabilities (in Percentage Points) of Perceived Discrimination. *Note:* The probabilities are predicted based on Model 2 of Table 2, with other covariates set at the sample means.

After controlling for covariates, compared with non-Hubei people, the odds of reporting greater perceived discrimination were 2.115 (= exp[0.749]) times higher for Hubei residents and 3.086 (= exp[1.127]) times higher for Hubei people living outside Hubei (permutation *p* value = .000 for both). Meanwhile, the odds of having greater perceived discrimination were 2.411 (= exp[0.880]) times higher for suspected/confirmed COVID-19 patients than for nonpatients (permutation *p* value = .000). These odds ratios (i.e., 2.115, 3.086, and 2.411) indicate a small- to medium-level effect size (Chen, Cohen, and Chen 2010). To facilitate understanding, we also present in Figure 4 the predicted probabilities of perceived discrimination (from never to often/always) by COVID-19 infection status (Panel A) and Hubeiness (Panel B).

#### Perceived discrimination and psychological distress

Table 3 shows that perceived discrimination during the Spring Festival was positively associated with psychological distress. Controlling for Hubeiness, COVID-19 infection status, and other covariates, the psychological distress score for people who rarely, sometimes, and often/always felt being discriminated against during the Spring Festival was 2.273, 4.366, and 7.351 points higher, respectively, than those who perceived no discrimination during this time (Model 3: permutation *p* value = .000 for all).

**Table 3.** Ordinary Least Squares Regressions Predicting Psychological Distress.

	Model 1		Model 2		Model 3	
	Coefficient	Permutation *p* Value	Coefficient	Permutation *p* Value	Coefficient	Permutation *p* Value
COVID-19 infection status						
Nonpatients						
Patients (suspected/confirmed)	4.304	.000	3.480	.000	2.501	.000
Prefer not to say	1.651	.001	.944	.062	.578	.247
Hubeiness						
Non-Hubei people						
Hubei residents	1.706	.000	1.559	.000	.794	.000
Hubei people living outside Hubei	1.194	.099	1.303	.071	.110	.881
Perceived discrimination						
Never						
Rarely					2.273	.000
Sometimes					4.366	.000
Often/always					7.351	.000
**Control variables**						
Female			–.068	.585	.053	.673
Age			–.056	.000	–.043	.000
Marital status						
Never married						
Married			–.486	.010	–.386	.040
Previously married			.303	.519	.237	.609
Presence of child						
No minor children						
Youngest child <6			.182	.281	.187	.271
Youngest child ages 6–17			–.208	.213	–.103	.548
Education						
Less than high school						
High school			–.306	.280	.028	.920
Junior college			–.058	.830	.229	.418
University or above			–.148	.592	.081	.768
Employment status prior to the outbreak						
Employed						
Unemployed			1.449	.000	1.151	.000
Not in the labor force			–.121	.434	.061	.693
Rural *hukou*			.092	.502	.181	.190
Monthly family income in 2019						
<5,000 yuan						
5,000–9,999 yuan			–.333	.037	–.336	.036
10,000–19,999 yuan			–.361	.044	–.399	.024
≥20,000 yuan			–.400	.071	–.486	.025
Self-rated health			–1.964	.000	–1.452	.000
Constant	7.014	.000	15.292	.000	11.489	.000

*Note: N* = 7,942. Interpretation of permutation *p* value: If 50 out of 10,000 permutations yield regression coefficients as large as the observed value, the probability that the actual coefficient could be the result of random sampling error is about .005.

Table 3 also reveals region-based and COVID-19 infection-status-based disparities in psychological distress irrespective of controls (Models 1 and 2). Holding other covariates constant, compared with non-Hubei people, the psychological distress score was 1.559 points higher for Hubei residents and 1.303 points higher for Hubei people living outside Hubei (permutation *p* values = .000 and .071, respectively). The psychological distress score was 3.480 points higher for suspected/confirmed patients than for nonpatients (permutation *p* value = .000).

Lastly, we compare Models 2 and 3 (Table 3) to examine whether perceived discrimination accounts for region-based and COVID-19 infection-status-based disparities in mental health. Specifically, the coefficient for Hubei residents reduces from 1.559 to .794 after controlling for perceived discrimination. Thus, perceived discrimination explains 49% (= [1.559 − 0.794] / 1.559) of the mental health disparity between Hubei residents and non-Hubei people. Perceived discrimination also mediates 92% of the mental health disparity between Hubei people living outside Hubei and non-Hubei people. Additionally, adding perceived discrimination reduces the coefficient for patients from 3.480 to 2.501, suggesting that perceived discrimination mediates 28% of the mental health disparity between suspected/confirmed COVID-19 patients and nonpatients.

### Interview Results

To complement our quantitative findings, the qualitative results in the following illustrate how the stigmatized groups perceived, experienced, and responded to stigma and discrimination during China’s COVID-19 outbreak.

#### Region-based stigma and discrimination

Our interviewees frequently mentioned stigma associated with Hubeiness. Indeed, because the COVID-19 outbreak began in Wuhan (the capital city of Hubei province), Wuhan people were sometimes the direct target of region-based stigma. For example, as Lin Mengqi (female, 34 years old) described:

At the beginning of the COVID-19 outbreak . . . many people thought it was caused by Wuhan people eating bats. I’ve seen many people attacking Wuhan people for this on Weibo [a Chinese social media platform similar to Twitter]. It’s region-based stereotyping (*diyu hei*). Accusations were flooding onto social media: “Why do you, Wuhan people, eat bats? Why do you, Wuhan people, make people throughout the country suffer from the outbreak?” After seeing this, you’d feel very sad, because you know that this is not true but all of a sudden, your life is no longer peaceful and instead, you’ve become a target of blame.

Lin’s account clearly exemplifies how different components in Link and Phelan’s (2001) theorizing coalesce to produce stigma. First, region-based differences were amplified, as evidenced by the multiple mentions of “Wuhan people” and the phrase of *diyu hei* (region-based stereotyping). Second, Wuhan people were stereotyped as “eating bats” and blamed for causing the outbreak. Third, a separation of them from us was evident (“you” have become other people’s “target of blame”). Combined, region-based labeling, stereotyping, and them/us separation led to perceived discrimination and loss of status. As a member of the stigmatized group, Lin Mengqi felt sad (and other negative emotions, as we describe in the following).

Lin’s account highlights the important role of social media in spreading and aggravating stigma. Other interviewees experienced more explicit discrimination in the form of rejection in sales or job applications. Song Zhengxiong (male, 42) was an entrepreneur who ran an online business in addition to his local store. We interviewed him on May 5, 2020, when few new cases were reported in Hubei, but discrimination was still present: “Online retail sales were pretty terrible. Especially a while ago, people were unwilling to buy things from Wuhan. . . . It was online communication, so, you know, people can be blunt and they expressed serious concerns about whether things from Wuhan would carry virus.” Similarly, Jiang Baiyu (male, 25) applied for several jobs in Shenzhen but did not get a single interview. He speculated that, “If a similarly qualified candidate was a Shenzhen person (*Shenzhen ren*) or a person from elsewhere, they [employers] would feel that candidate was better than someone from Wuhan. . . . I feel that, as soon as I said I was a Wuhan person (*Wuhan ren*), their interest dissipated quickly.”

Hubei people living outside Hubei were also target of stigma and discrimination. When asked if she had any closing remarks, Xu Nana (female, 30) recounted discriminatory experiences of her friend’s family in Guangzhou; among other things, her quote reveals how vehicle license plates became a public indicator of Hubeiness:

I still feel that people, more or less, have some intentional and unintentional prejudice against Wuhan or Hubei people. . . . I hope that they don’t do this anymore. . . . I have a good friend who drove to Guangzhou during the outbreak. . . . Their license plate number begins with “È A,” indicating Wuhan origin, so all their neighbors knew that they were from Wuhan. When they took their children outdoors, their neighbors would all go home. The parking spots next to their car were always empty. . . . Anyhow, they felt that they were treated somewhat differently.

Our interviews also reveal the critical role of power in producing region-based stigma. Wang Li, a woman in her 30s, went to Hainan with her family for vacation before the 2020 Spring Festival. Their flight back to Wuhan was cancelled because of Wuhan’s sudden coronavirus lockdown. They could not find a place to live—rental agents turned them down after finding out that they came from Hubei. Fortunately, her friend had an empty apartment in Shenzhen, where her family flew to on Chinese New Year’s Eve and stayed until late March 2020 before going back to Wuhan. While staying in Shenzhen, Wang received many phone calls from authorities in Hainan and Shenzhen to inquire about her family’s travel history, health status, and current situation. She explained to the interviewer that there was a national network for epidemic monitoring (*quanguo liandong*), so travelers with a Hubei *hukou* or citizen identity card could be easily tracked down. When they arrived in Shenzhen, local *shequ* officials quickly came to their door and asked her family to go through home quarantine. Later, *shequ* officials even put a seal on their door, put up a notice with the start and end date of their quarantine (as she put it, “all neighbors could see it”), and installed a camera to monitor them. The exercise of institutional power was evident throughout: Without power, it would have been impossible to know the phone numbers and travel histories of people who were associated with Hubei or to implement contact tracing and mandatory quarantine in ways that made their *Hubeiness* label public.

#### Disease-associated stigma and discrimination

Our interviewees who were once suspected or confirmed to have COVID-19 commonly voiced disease-associated stigma. Gu Jia (female, 36) was a confirmed patient and once quarantined in a facility along with other COVID-19 patients. They formed an online chat group. In the following, Gu comforted one member of the chat group who experienced discrimination:

People are very unfriendly to COVID-19 patients and may ignore you or deliberately keep a distance from you. . . . Just two days ago, I comforted an older lady in a chat group consisting of people from the centralized quarantine facility. She said, “I was a very popular neighbor before, and all the people in my neighborhood liked talking with me a lot. But now, when I go out, they intentionally avoid me. I can’t take it.” Things like this could have huge psychological impacts on us.

Another confirmed patient, Shen Yan (female, 34), encountered discrimination firsthand in her neighborhood. At the peak of the outbreak, when medical resources were extremely scarce, she aired her case online (including her name, phone number, and WeChat ID) in a desperate effort to find a hospital bed. After her hospital discharge, her neighbors demanded public disclosure of her private information in her *shequ*’s chat group:

People in my neighborhood knew that I once sought help online. . . . Even after my home isolation following hospital discharge, some neighbors were still hunting “COVID-19 witches” in our *shequ* WeChat group and requesting the property management company to announce our exact unit number—things like this. . . . We don’t want to harm others either. I feel very uncomfortable psychologically when being discriminated against by others.

Discriminatory treatment can also come from relatives. Bai He (female, 21) was once a confirmed COVID-19 patient. She went to visit her relatives in mid-April 2020 after she was recovered. She shared that her uncle was “hostile” to her and that she felt “hurt”:

My cousin who is about the same age as me sat beside me. She wouldn’t wear a mask because she didn’t think I was infectious. But her father purposely scolded her for not wearing a mask. . . . I was very embarrassed and sad. Later, my aunt gave me snacks to eat. I deliberately went away, going upstairs to have the snacks by myself, but he came upstairs to scold me for not being precautious. . . . I was wearing a mask all the time when I was talking with them. Moreover, I intentionally wore an N95 mask, exactly because I was very worried that they would be concerned. But he still said things like that. . . . It really hurts.

In addition to *confirmed patient*, *suspected patient* was another state-endorsed label to classify populations. Suspected patients also experienced disease-associated stigma. For example, before the Spring Festival, Wan Ke, a man in his 20s, left Wuhan and went to his home village nearby. He had a fever and was publicly labeled as a suspected patient:

People around you would treat you differently. They would also deliberately alienate you or isolate you. . . . The staff in my *shequ* . . . they came and told me that I was a suspected COVID-19 patient. They used a speaker to announce my name and address in the village, broadcasting that I had symptoms that would classify me as a suspected patient.

The practices of labeling, stereotyping, and them/us separation to produce disease-associated stigma were evident in Wan’s recount:

Staff from our *shequ* put a door seal and wrote down “Confirmed COVID-19 Patient, Stay Clear” in extra-large font. The moment there were suspected or confirmed cases, the cadre [village official] would take the lead and act so dramatic! They wore protective clothes and goggles, as if they were dealing with a biochemical-viral fallout, and as soon as they got into patients’ homes, they sanitized everywhere with alcohol spray.

Because of the very public stigma-generating process, Wan said that all villagers were aware of and gossiping about those suspected and confirmed patients. The practices described by Wan may be extreme, but almost every interviewee in our study knew whether their neighborhood had suspected or confirmed COVID-19 cases and if so, how many and which building they lived in. This was because *shequ* were required to announce neighborhood-level COVID-19 information to residents. In short, similar to how Hubei people were treated, suspected and confirmed patients went through labeling, stereotyping (as virus carriers, often well after recovery), them/us separation, and discrimination in a power structure, revealing how stigma was linked to state interventions to curb the spread of COVID-19.

#### Stigma, discrimination, and psychological distress

According to Lin Mengqi, region-based stigma that went rampant on social media made her feel falsely accused and was a major source of her psychological distress:

Being attacked was very heartbreaking . . . very heartbreaking. . . . Let me put it this way, in fact, during the first half month of the COVID-19 outbreak, my psychological distress didn’t come from how risky the outbreak made me feel but from how wronged I felt during that time.

Similar negative emotions were shared among other interviewees, including COVID-19 patients, as shown by the earlier quotes from Gu Jia and Bai He. COVID-19-related stigma may understandably originate from fear, but the psychological consequences are palpable. As Shen Yan put it, “I think the discrimination in my *shequ* might stem from some sort of fear and panic. Even though I can understand it, I still feel a little uncomfortable in my heart.”

Overall, our interviewees expressed many adverse psychological effects of experiencing region-based or disease-associated stigma and discrimination, describing their emotions as “not good for sure,” “feeling our world collapsed,” “very upset,” “quite depressed,” “harmed,” and “angry.”

#### Coping strategies

Our interviews also reveal ways in which the stigmatized groups coped with stigma and discrimination. One coping strategy was secrecy (i.e., keeping their stigmatized status secret; Link et al. 1989). Wang Li used this strategy in Hainan when she was looking to rent a place for her family to live. However, such strategy was not effective in China, given the public nature of labeling and the authorities’ access to private information (facilitated by the national network for epidemic monitoring). A more common coping strategy adopted by our interviewees was to “withdraw from social contacts that they perceive as potentially rejecting” (Link et al. 1989:400). For example, Jiang Baiyu’s mom, once a confirmed patient, avoided going out and socializing after hospital discharge. Several interviewees, after mentioning the stigma associated with Hubeiness, said that they would avoid nonessential travel outside Hubei. This can be seen in our interview with Ye Zhihao (male, 29) on May 3, 2020. When asked about restrictions on leaving Wuhan, he said:

If you have a green code [a digital QR code on smartphone], you can leave the city. However, people outside Hubei tend to discriminate against people with Hubei *hukou*. . . . I haven’t been out of town, but I have heard of it. . . . So I won’t travel outside Hubei. I wouldn’t leave Hubei except under special circumstances.

Another strategy used was education, providing information to counter stereotypes (Link et al. 1989). A few interviewees mentioned that they were waiting for credible evidence (e.g., scientific research) on the origin of the virus to prove it wrong to stigmatize Hubei people. Others attempted to educate people who stigmatized “through actions.” Peng Hu (male, 45) was a volunteer during the outbreak. When asked why he became a volunteer, he said:

When the outbreak began, many people attacked Hubei people online, saying that Hubei caused the outbreak. I wanted to show them through my actions that, although we were victims, we had been working hard to get through this.

In addition to volunteer work in the community, he also engaged in challenging (direct and active confrontation of stigmatizing behavior; Link and Phelan 2013), such as talking back online, replying to social media posts, and commenting that Hubei people were not the virus and that the outbreak was not their fault. Nevertheless, he admitted that he was distressed. The strategy of challenging was also used by Lin Mengqi and several others; they spent a lot of time online arguing against people who expressed “region-based stereotyping” (*diyu hei*) views. According to Lin, she “can’t help” confronting those stigmatizing social media posts.

Some interviewees coped by talking about their feelings with “experientially similar others” (i.e., people who have been through similar experiences; Thoits et al. 2000). As illustrated in Gu Jia’s narratives, she and other COVID-19 patients shared their experiences and feelings and supported each other in the chat group. “I think talking about it was a very good way to let go of unhappiness and negative energies,” Gu commented. Likewise, nonpatient interviewees who nevertheless experienced region-based stigma coped by chatting with their friends or colleagues. Providing and receiving empathic understanding with experientially similar others facilitated effective coping.

## Discussion

The COVID-19 pandemic has transformed every facet of society. In addition to directly affecting morbidity and mortality, it has given rise to social problems that reverberate to shape public health, such as stigma and discrimination. This mixed-methods research examines the stigma that arose during China’s COVID-19 outbreak, the heightened discrimination and psychological distress perceived by the stigmatized groups, and how the stigmatized groups coped with discrimination and the resulting psychological distress. Given the still ongoing COVID-19 pandemic, our findings have important implications for population mental well-being worldwide.

First, our research illustrates that disease-associated and culturally created categories become important bases for distinguishing “us” from “them” in a public health crisis and lead to two types of stigma. In line with research on diseases and stigma (Joffe 1999; Parker and Aggleton 2003), our interview data show the emergence of stigma directly related to COVID-19, thereby providing supportive evidence that some stigma may reflect one’s desire to “keep people away” (Phelan et al. 2008). Additionally, rooted in China’s preexisting social practice that uses region to categorize human differences (Eberhard 1965; Moser 2019), region became another source of stigma during China’s COVID-19 outbreak. The growing fear of the virus has quickly turned into the fear toward and discrimination against people from the hardest hit province and people who are socially associated with the epicenter, Hubei province. Our survey experiment, indeed, demonstrates the strong stigma attached to Hubeiness. This region-based stigma in China reveals that the sources of stigma can often be traced back to preexisting social contexts, thereby highlighting the social construction nature of stigma.

In addition to identifying the sources of stigma, our research uncovers the social and institutional forces in stigma-generating processes. Interview data show that stigma spreads not only through in-person interaction but also via social media. In fact, moral accusations on social media played an important role in labeling and stereotyping Hubeiness and separating Hubei from non-Hubei people. Furthermore, institutional forces in the form of state power are essential to producing disease-associated and region-based stigma. Facilitated by technology (WeChat), *shequ* practices that relied heavily on state-endorsed labels such as *confirmed patients* and *suspected patients* turned out to be key to generating disease-associated stigma. Also, the *hukou* system that has long served as a tool of migration control and massive surveillance facilitated epidemic monitoring yet also labeling, tracing, isolating, and ultimately stigmatizing Hubei people who were outside Hubei. Therefore, although the Chinese government “has done everything in its power to reduce infections to the minimum” (State Council Information Office 2020:32), public health measures used to curb the spread of COVID-19 are not without costs.

Second, survey results show that perceived discrimination is associated with greater psychological distress and mediates much of the relationship between Hubeiness—or COVID-19 infection status—and psychological distress. These findings showcase one central contribution of this research, that is, discrimination can be damaging to health. Not only do people who are geographically or socially affected by the disease face higher risk of exposure to COVID-19, but they also have to contend with the additional threat of stigmatization and discrimination. In view of the urgent need of psychological well-being recovery postpandemic, our results point to targeting discrimination as a possibly effective avenue. Our research therefore contributes much needed knowledge to designing pandemic recovery interventions for China and elsewhere.

At the time of our writing, confirmed COVID-19 cases have exceeded 100 million globally, not counting those who show suspected symptoms. Given the large number of people affected and the possibility that stigma exists well after recovery, the psychological healing process has a long way to go. Meanwhile, it remains an open question how stigma evolves over time and across societies. For example, as the outbreak gradually becomes under control in China, will stigma associated with Hubeiness disappear, linger around, or in the worst-case scenario, become institutionalized? As new epicenters emerge, will stigma and discrimination shift their target? Longitudinal and cross-national data are needed to elucidate the dynamic, culturally embedded stigma processes that depend on the ways laws, social services, and the justice system are structured. These research endeavors will be imperative for designing effective interventions to reduce stigma and for minimizing social and public health impacts of the ongoing pandemic.

Third, our in-depth interviews have made visible the voices of the stigmatized. Stigmatized groups are not merely passive victims being labeled and discriminated against; they actively respond to and cope with stigma in various ways. In addition to the strategies identified in previous research (Link et al. 1989; Link and Phelan 2013)—such as secrecy, withdrawal, education, and challenging—we find that some interviewees coped by talking with “experientially similar others” (Thoits et al. 2000). The scale of COVID-19 likely makes it possible to identify others with similar experiences. Not all strategies, however, are equally effective. Given the public forms of labeling and the authorities’ access to private information, secrecy, for example, was not found by our interviewees to be successful in managing the stigma they encountered.

Our research has limitations. First, our survey was based on an online, nonrandom sample, which limits our ability to generalize. When compared with a nationally representative survey, our sample has similar distributions of marital status and rural *hukou* but overrepresents Hubei residents, younger adults, those with more education, and women (with the gender difference being very small; see Appendix Section 2 in the online version of the article). Although we control for characteristics that may have affected survey participation, an underrepresentation of older adults and those with less access to the Internet suggests that we may overestimate the extent of perceived discrimination (given these people’s less exposure to discriminatory remarks on social media). As for the relationship between perceived discrimination and psychological distress, a representative sample would be unlikely to change our findings given that the discrimination–distress relationship does not vary by education or age (for more details, see Appendix Section 3 in the online version of the article).

Second, the sample sizes for some groups (e.g., Hubei people living outside Hubei) were small, which may affect the stability of some estimates. We alleviate this issue by using nonparametric permutation tests for inference. A larger sample size for these groups would likely yield more significant results with enhanced estimation precision (for more discussion, see Appendix Section 4 in the online version of the article). Third, our measure of perceived discrimination did not specify the basis or the form of discrimination. We control for major sources of discrimination in the Chinese context (e.g., gender, *hukou*); therefore, the estimates for COVID-19 infection status and Hubeiness indicate perceived discrimination net of these alternative sources. Additionally, the significantly higher level of perceived discrimination among Hubeiness compared with non-Hubeiness is unlikely to result from attributes other than region because other types of discrimination (e.g., gender discrimination) would happen for both groups to a similar extent. Our qualitative data also suggest that region and COVID-19 infection status were two major sources of discrimination during China’s COVID-19 outbreak. Nevertheless, future quantitative assessment may benefit from more detailed measures of perceived discrimination. Fourth, our survey is cross-sectional, which limits our ability to speak to causal ordering. Future longitudinal research could shed more light on the dynamic relationships between stigma, perceived discrimination, and psychological distress.

In sum, disasters and epidemics make visible the social distribution of vulnerability and the deep-rooted problems of society. Our findings indicate that the COVID-19 outbreak created region-based and disease-associated stigma in China, with people living in or originating from Hubei and those suspected or confirmed to have COVID-19 at higher risk of being stigmatized and discriminated against. Perceived discrimination, in turn, is associated with greater psychological distress, as shown by our survey and interview data. Interview data also illuminate institutional forces underlying stigma production and coping strategies adopted by the stigmatized groups. To aid postcrisis well-being recovery, we advise more public policies oriented toward addressing the collateral human cost of the pandemic such as stigma and discrimination.

## Supplemental Material

sj-pdf-1-hsb-10.1177_00221465211040550 – Supplemental material for Stigma, Perceived Discrimination, and Mental Health during China’s COVID-19 Outbreak: AMixed-Methods Investigation Supplemental material, sj-pdf-1-hsb-10.1177_00221465211040550 for Stigma, Perceived Discrimination, and Mental Health during China’s COVID-19 Outbreak: AMixed-Methods Investigation by Wen Fan, Yue Qian and Yongai Jin in Journal of Health and Social Behavior
