# Supplementary material for: Stigma, Perceived Discrimination, and Mental Health during China’s COVID-19 Outbreak: A Mixed-Methods Investigation
Source: J Health Soc Behav. 2021 Oct 4;62(4):562–81. doi: 10.1177/00221465211040550 (PMC8637388; doi:10.1177/00221465211040550)

Journal of Health and Social Behavior

OFFICIAL JOURNAL OF THE AMERICAN SOCIOLOGICAL ASSOCIATION

ONLINE SUPPLEMENT

to article in

Journal of Health and Social Behavior

Stigma, Perceived Discrimination, and Mental Health during China's COVID-19 Outbreak: A Mixed-methods Investigation

Wen Fan*
Boston College

Yue Qian*
University of British Colombia

Yongai Jin
Renmin University of China

*** Wen Fan and Yue Qian share first authorship.**

1. Wording of the survey experiment items

In the survey, respondents were presented two social distance questions:

- Nowadays, how do you think most people in this society would feel about having … enter the city they live in?
- Nowadays, how do you think most people in this society would feel about having … enter the neighborhood they live in?

Through randomization, about half of the respondents were asked about Sichuan people (*Sichuan ren*) and the other half were asked about Hubei people (*Hubei ren*).

We used "most people" in the wording of our survey experiment items because social desirability is a potential concern in attitudinal studies, that is, people may not admit their true attitudes when it comes to prejudice or bias. We thus adopted a strategy known as "projective tests" in psychology—instead of directly asking one's own reactions, respondents were asked to describe their attitudes of what most people in society would feel (Cook and Selltiz 1964; Vargas, Hippel, and Petty 2004).

This strategy is especially useful for our study for the following reasons. First, the "projective" nature of the wording allows us to alleviate social desirability bias to capture respondents' underlying disposition. Second, the social distance measures enable us to assess the degree to which Hubei people were discriminated against in the society. Specifically, to Hubei people, the measures reflect the degree of social distance (and discrimination) they perceived from other people; to non-Hubei people, the measures reflect at what social distance they wanted to keep away from Hubei people. Combined, responses to the social distance measures reflect the public stigma associated with Hubeiness in society. Lastly, Link used similar measures to capture stigma and beliefs about discrimination in his research (1987:102): "The main independent variable in this study was measured by 12 items (see Appendix) that were written to assess the extent to which an individual believes most people will devalue or discriminate against a psychiatric patient..." Moreover, in Link et al.'s (1989) seminal research, they drew on samples of community residents without psychopathology and mental patients to show that both patients and community residents believe that "most people" will reject mental patients. We have thus taken a similar approach to assess stigma in a different context (i.e., the context of China's COVID-19 outbreak).

Given that Hubei residents were oversampled in our study, to further make sure that our measures do not merely reflect how Hubei residents thought about themselves, we compared results between Hubei people and non-Hubei people. The patterns are similar across non-Hubei people, Hubei people, and the combined sample (Appendix Figures 1–3 on pp.9–11 of this document). The findings thus support our argument that the social distance measures capture the public stigma around "Hubeiness", and that both Hubei and non-Hubei people recognize the stigma.

2. How the sample compares to the general population

We have compared our sample with a nationally representative sample from the 2017 Chinese General Social Survey (CGSS), which is its most recent wave with publicly available data.

Results in Appendix Table 1 (p.5 of this document) show that compared with the nationally representative CGSS sample, our sample overrepresents Hubei residents and women; respondents in our sample are also younger and more educated on average. Our sample, however, does not differ from the nationally representative sample in the distribution of marital status or rural *hukou*, holding other demographic variables constant. Note that although our sample appears to overrepresent women statistically speaking, the actual difference in the gender distribution between our sample and the CGSS sample is very small in magnitude: holding other covariates constant, the share of women is 48% in the CGSS sample and 52% in our sample; in terms of raw distributions, female respondents account for 49% of the CGSS sample, and 50% of our sample.

Additionally, given that our survey was based on an online sample, individuals with less access to the internet were underrepresented. According to the 2020 Report on Internet Development in China, the internet penetration rate reached 65 percent in China by March 2020. [1] Underrepresenting those with less access to the internet suggests that we may have overestimated the extent of perceived discrimination (given these people's less exposure to discriminatory remarks on social media).

3. Whether age and education moderate the relationships between Hubeiness/COVID-19 infection status/perceived discrimination and mental health

Considering that our sample is younger and more educated compared to the general population, we control for age and educational attainment in the statistical models presented in our paper. In supplementary analysis, we examined age and education as moderating variables by adding interaction terms between age (or education) and Hubeiness, COVID-19 infection status, or perceived discrimination, in models predicting psychological distress. None of the interaction terms were significant at the .05 level (see Appendix Tables 2 and 3 on pp.6–8 of this document). Thus, our findings about the relationships between mental health and Hubeiness, COVID-19 infection status, or perceived discrimination are unlikely to change even with a representative sample with respect to age and education.

4. Small sample sizes for certain groups

We acknowledge that the sample sizes for some groups are relatively small. In terms of COVID-19 infection status, it is worth noting that the share of confirmed or suspected COVID-19 cases was very small at the population level. For example, while China's population is approximately 1.4 billion, the cumulative number of confirmed COVID-19 cases was 82,862 as of the last day of our survey (April 29, 2020), according to the National Health Commission.[2] The number and share of suspected cases are more difficult to gauge, given the lack of official statistics, though the share is likely small as well given China's population size. Combined, it is reasonable that confirmed/suspected patients make up only 1.46% of our sample.

In terms of Hubei people living outside Hubei, we compared our sample with national statistics, and found that the proportions were of similar magnitudes: Hubei people living outside Hubei

[1] http://www.cnnic.cn/hlwfzyj/hlwxzbg/hlwtjbg/202004/t20200428_70974.htm.
[2] http://www.nhc.gov.cn/xcs/yqtb/202004/ce78f5575e0d4ef0b4543eb072acebc3.shtml.

make up 0.7% of our sample, compared with about 0.5% using China's 2015 mini census data. Thus, Hubei people living outside Hubei are a small group even at the population level.

Although the relative share is small, the absolute number is not tiny (116 COVID-19 cases and 59 Hubei people living outside Hubei in our sample), which are considered to be reasonable by statisticians. For example, Pett (1977) and Salkind (2004) defined 30, whereas Warner (2008) used 20, as a cutoff point for a small sample. The use of the non-parametric permutation method also helps address the small sample size issue. The findings from our quantitative data for these groups also align well with our qualitative findings, which illustrate how both region-based and disease-associated stigma and discrimination emerged during China's COVID-19 outbreak and affected the psychological distress of the stigmatized groups.

References:

Cook, S. W., & Selltiz, C. (1964). A multiple-indicator approach to attitude measurement. *Psychological Bulletin, 62*(1), 36–55.

Link, B. (1987). Understanding labeling effects in the area of mental disorders: An assessment of the effects of expectations of rejection. *American Sociological Review, 52*(1), 96–112.

Link, B. G., Cullen, F. T., Struening, E., Shrout, P. E., & Dohrenwend, B. P. (1989). A modified labeling theory approach to mental disorders: An empirical assessment. *American Sociological Review, 54*(3), 400–423.

Pett, M.A. (1997). *Nonparametric statistics for health care research: Statistics for small samples and unusual distributions*. Thousand Oaks, CA: Sage Publications.

Salkind, N.J. (2004). *Statistics for people who (think they) hate statistics*. 2nd ed. Thousand Oaks, CA: Sage Publications.

Vargas, P. T., Von Hippel, W., & Petty, R. E. (2004). Using partially structured attitude measures to enhance the attitude-behavior relationship. *Personality and Social Psychology Bulletin, 30*(2), 197–211.

Warner, R.M. (2008). *Applied statistics: From bivariate through multivariate techniques*. Thousand Oaks, CA: Sage Publications.

Appendix Table 1. Binary Logit Regression Models Predicting the Log-Odds of Respondents from the Sample Used in the Current Study Relative to the 2017 Chinese General Social Survey Sample

	Coef.		SE
Hubei Province	3.968	***	(0.084)
Female	0.150	**	(0.052)
Age	-0.048	***	(0.003)
Education			
Less than high school			
High school	1.936	***	(0.093)
Junior college	2.585	***	(0.098)
University or above	2.800	***	(0.096)
Marital status			
Never married			
Married	-0.036		(0.066)
Previously married	-0.049		(0.155)
Rural *hukou*	-0.046		(0.058)
Constant	-1.697	***	(0.141)

Note: Coef. = Coefficient. SE = Standard Error. The size of the 2017 Chinese General Social Survey sample = 12,546; the size of the sample used in the current study = 7,942. Weights provided by the Chinese General Social Survey are used, and respondents from the sample used in the current study are each assigned a weight of 1.

*** $p<0.001$, ** $p<0.01$, * $p<0.05$.

Appendix Table 2. OLS Regression Models Predicting Psychological Distress, Adding Interaction Terms with Age

	Coef.	Permutation *p*-value	Coef.	Permutation *p*-value
COVID-19 infection status				
Non-patients				
Patients (suspected/confirmed)	1.502	0.422	-0.184	0.917
Prefer not to say	-0.206	0.899	-1.500	0.352
Hubeiness				
Non-Hubei people				
Hubei residents	0.803	0.071	0.555	0.217
Hubei people living outside Hubei	-1.456	0.580	-0.419	0.875
Perceived discrimination				
Never				
Rarely			1.718	0.000
Sometimes			4.750	0.000
Often/always			6.653	0.000
Age	-0.076	0.000	-0.056	0.000
COVID-19 infection status * Age				
Patients (suspected/confirmed) * Age	0.064	0.275	0.088	0.126
Prefer not to say * Age	0.036	0.465	0.065	0.183
Hubeiness * Age				
Hubei residents * Age	0.025	0.077	0.008	0.595
Hubei people living outside Hubei * Age	0.090	0.275	0.016	0.852
Perceived discrimination * Age				
Rarely * Age			0.018	0.234
Sometimes * Age			-0.013	0.484
Often/always * Age			0.023	0.450
Other Control Variables	Yes		Yes	

Note: N = 7,942. Coef. = Coefficient. The models also control for female, education, marital status, presence of child, employment status prior to the outbreak, rural *hukou*, monthly family income in 2019, and self-rated health.

Interpretation of permutation *p*-value: If 50 out of 10,000 permutations yield regression coefficients as large as the observed value, the probability that the actual coefficient could be the result of random sampling error is 0.005.

Appendix Table 3. OLS Regression Models Predicting Psychological Distress, Adding Interaction Terms with Education

	Coef.	Permutation *p*-value	Coef.	Permutation *p*-value
COVID-19 infection status				
Non-patients				
Patients (suspected/confirmed)	2.170	0.301	0.998	0.636
Prefer not to say	1.571	0.260	0.934	0.503
Hubeiness				
Non-Hubei people				
Hubei residents	1.838	0.000	1.264	0.017
Hubei people living outside Hubei	0.604	0.854	-0.336	0.920
Perceived discrimination				
Never				
Rarely			2.777	0.000
Sometimes			5.065	0.000
Often/always			6.588	0.000
Education				
Less than high school				
High school	0.340	0.512	0.819	0.159
Junior college	0.291	0.566	0.819	0.148
University or above	-0.154	0.754	0.608	0.274
COVID-19 infection status * Education				
Patients (suspected/confirmed) * High school	-1.107	0.664	-0.500	0.846
Patients (suspected/confirmed) * Junior college	0.958	0.672	1.260	0.586
Patients (suspected/confirmed) * University or above	2.233	0.315	2.230	0.323
Prefer not to say * High school	-0.059	0.974	0.196	0.906
Prefer not to say * Junior college	-1.614	0.350	-1.025	0.553
Prefer not to say * University or above	-0.683	0.674	-0.466	0.774
Hubeiness * Education				
Hubei residents * High school	-0.991	0.103	-0.834	0.168
Hubei residents * Junior college	-0.468	0.439	-0.620	0.296
Hubei residents * University or above	0.055	0.924	-0.303	0.593
Hubei people living outside Hubei * High school	1.090	0.772	1.159	0.757
Hubei people living outside Hubei * Junior college	0.780	0.824	0.012	0.996
Hubei people living outside Hubei * University or above	0.707	0.837	0.607	0.856
Perceived discrimination * Education				
Rarely * High school			-0.282	0.660
Rarely * Junior college			-0.434	0.489
Rarely * University or above			-0.688	0.254
Sometimes * High school			-1.020	0.190
Sometimes * Junior college			-0.464	0.533
Sometimes * University or above			-0.824	0.245
Often/always * High school			0.204	0.858
Often/always * Junior college			1.070	0.317
Often/always * University or above			0.860	0.379

Other Control Variables	Yes	Yes

Note: *N* = 7,942. Coef. = Coefficient. The models also control for female, age, marital status, presence of child, employment status prior to the outbreak, rural *hukou*, monthly family income in 2019, and self-rated health. Interpretation of permutation *p*-value: If 50 out of 10,000 permutations yield regression coefficients as large as the observed value, the probability that the actual coefficient could be the result of random sampling error is 0.005.

Appendix Figure 1. Percentage Distribution of the Responses to Social Distance Measures in Survey Experiment, Responses from Non-Hubei People

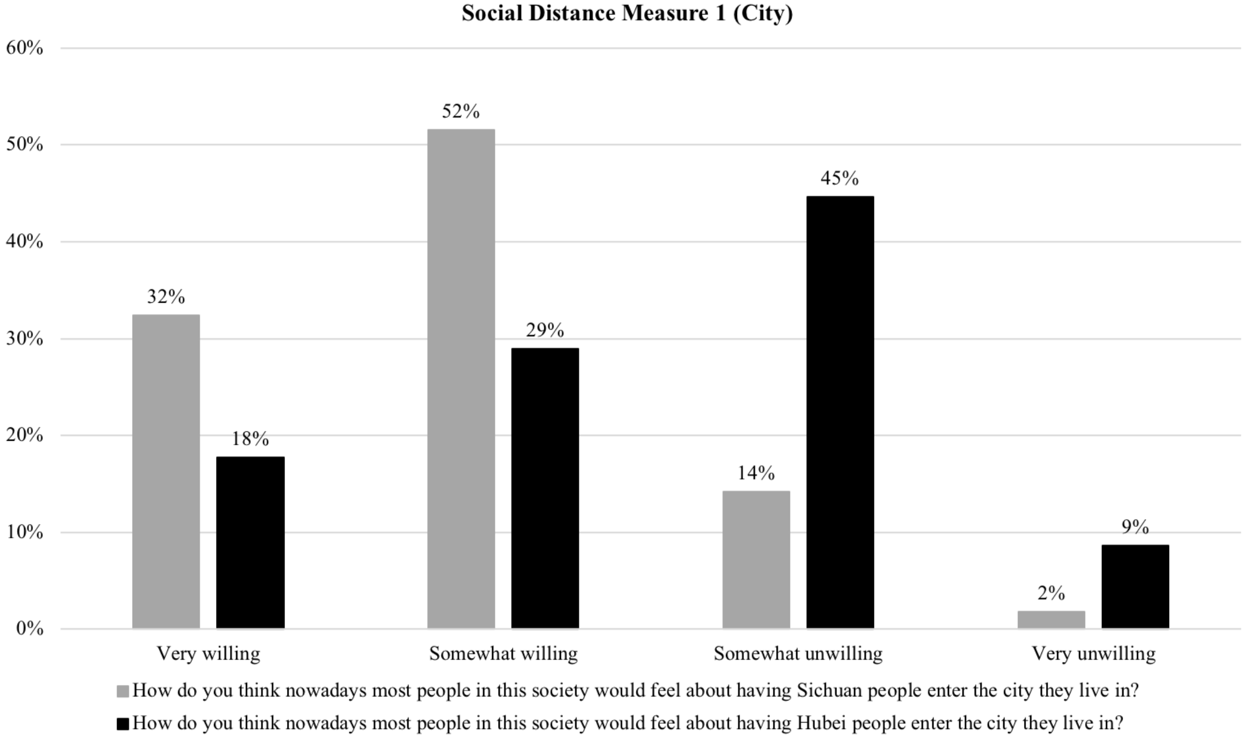

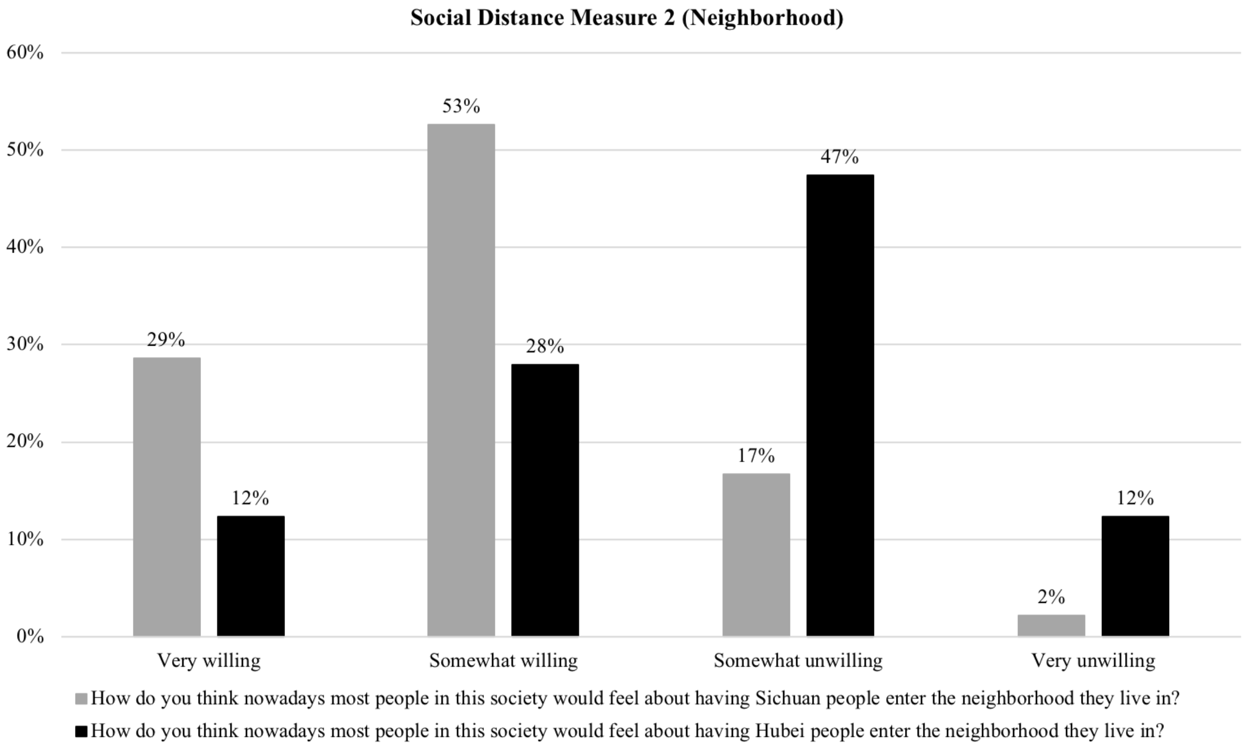

Appendix Figure 2. Percentage Distribution of the Responses to Social Distance Measures in Survey Experiment, Responses from Hubei People

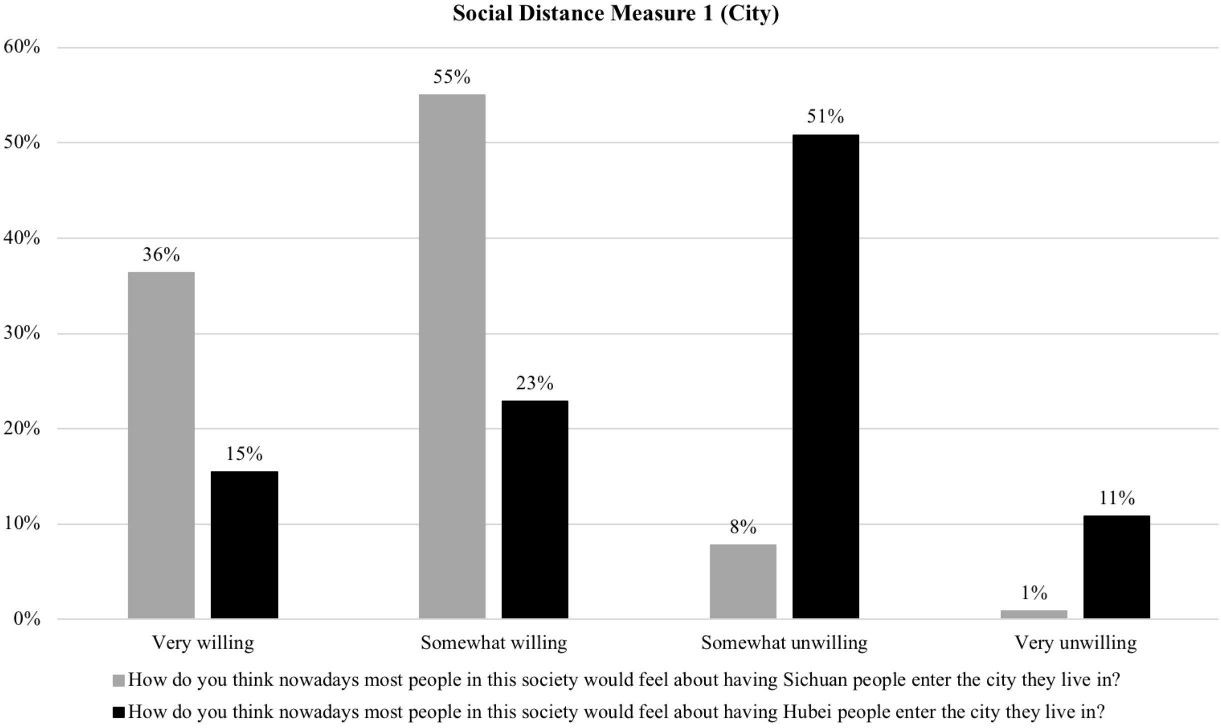

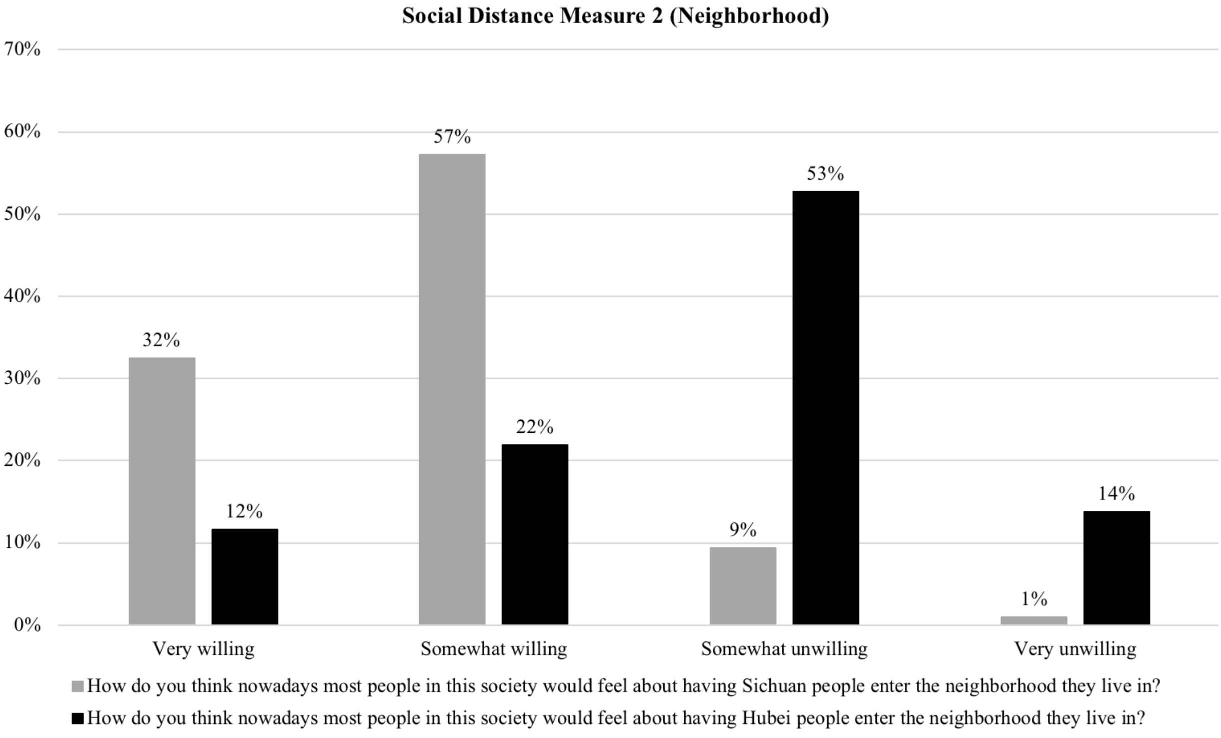

Appendix Figure 3. Percentage Distribution of the Responses to Social Distance Measures in Survey Experiment, Responses from All Respondents (Same as Figure 3 in the Main Text)

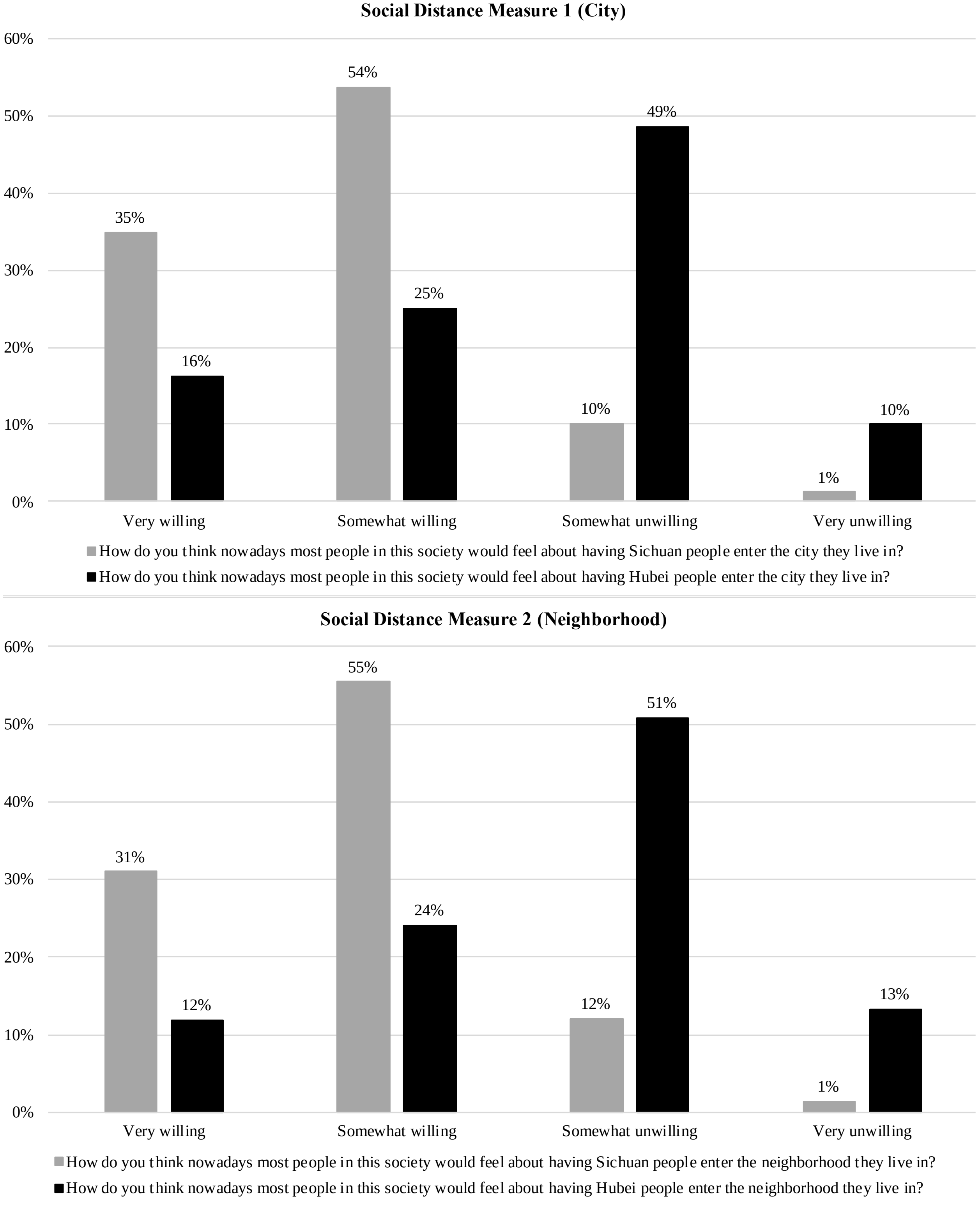

Supplement: sj-pdf-1-hsb-10.1177_00221465211040550 – Supplemental material for Stigma, Perceived Discrimination, and Mental Health during China’s COVID-19 Outbreak: AMixed-Methods Investigation [file sj-pdf-1-hsb-10.1177_00221465211040550.pdf]
